# Clinical Correlations of Novel Autoantibodies in Patients with Dry Eye

**DOI:** 10.1155/2019/7935451

**Published:** 2019-01-13

**Authors:** Sezen Karakus, Alan N. Baer, Esen K. Akpek

**Affiliations:** ^1^Ocular Surface Diseases and Dry Eye Clinic, The Wilmer Eye Institute, Johns Hopkins University, Baltimore, Maryland, USA; ^2^The Johns Hopkins Jerome L. Greene Sjögren's Syndrome Center, Baltimore, MD, USA

## Abstract

**Background:**

Diagnostic criteria for Sjögren's syndrome (SS) are continually being updated in pursuit of more precise and earlier diagnosis to prevent its complications. Owing to the high rate of false negative traditional serological markers, the need for better serological testing remains.

**Objective:**

To investigate the clinical significance of three recently discovered novel autoantibodies, anti-salivary gland protein 1 (SP1), anti-carbonic anhydrase 6 (CA6), and anti-parotid secretory protein (PSP), in a cohort of dry eye patients with suspected underlying inflammatory/autoimmune disease.

**Methods:**

Medical records of 136 patients with a primary diagnosis of dry eye who underwent laboratory testing between April 2014 and July 2017 were reviewed retrospectively. Data regarding demographic information, ocular and systemic symptoms, previous medical diagnoses, serological test results, and minor salivary gland biopsy results were collected. Dry eye evaluations included tear osmolarity, Schirmer test without anesthesia, conjunctival lissamine green staining, and corneal fluorescein staining in the order listed here.

**Results:**

Of the 136 patients, 9 (9/136, 6.6%) presented with a history of SS, and 9 additional patients (9/127, 7%) received a new diagnosis of SS as a result of evaluations. Fifty-six patients (56/136, 41%) tested positive for at least one of the novel autoantibodies. Fifty-four percent (6/11) of patients with primary SS who underwent the novel serological testing had a positive anti-PSP. Of those, 2 (2/11, 18%) had negative traditional serology and had to undergo minor salivary gland biopsy for definitive diagnosis. Anti-CA6 was associated with increased corneal and conjunctival staining after adjusting for age, sex, and other serologic markers (HR = 1.5, 95% CI = 1.20-1.97, and *p* = 0.009 and HR = 1.4, 95% CI = 1.04-1.76, and *p* = 0.02, respectively).

**Conclusions:**

This cross-sectional study demonstrated that anti-CA6 is seen in patients with severe aqueous-deficient dry eye. Whether these patients have an early stage of SS or a different type of autoimmune condition may be determined through longitudinal studies.

## 1. Introduction

Dry eye is a highly prevalent disease that affects up to 50% of the population worldwide [1]. Although dry eye is recognized as a multifactorial disease of the tears, inflammation has been identified as a key element in the pathogenesis [2]. About half of the patients with clinically significant dry eye have an underlying systemic inflammatory/autoimmune disease [3]. It is relevant to recognize an underlying autoimmune process, such as Sjögren's syndrome (SS), since timely diagnosis with adequate treatment can prevent possible ocular and/or systemic complications [3–6]. Approximately 1/10 patients with clinically significant dry eye has underlying SS. However, diagnosis is usually severely delayed largely due to a lack of awareness and the complexity of patient symptoms and signs [3–6]. A previous report from our center demonstrated that half of the SS patients with a vision-threatening ocular finding did not have an established diagnosis at the time of the presentation, despite having evidence of significant systemic manifestations [5]. Therefore, a high index of suspicion is necessary to recognize the disease earlier and prevent possible complications.

According to the 2012 American College of Rheumatology (ACR) classification criteria, significant dry eye must be present with either positive serology [anti-SSA and/or anti-SSB or a combination of rheumatoid factor (RF) and antinuclear antibody (ANA) at a titer ≥ 1 : 320] or a positive minor salivary gland biopsy to allow a diagnosis of SS [7]. Recently, classification criteria for SS have been updated and approved for the first time by both the American College of Rheumatology (ACR) and the European League Against Rheumatism (EULAR) [8]. The new set of criteria includes 5 items: (1) focal lymphocytic sialadenitis with focus score ≥ 1, (2) anti-SSA positivity, (3) ocular staining score (OSS) ≥ 5 at least in one eye, (4) Schirmer test ≤ 5 mm at least in one eye, and (5) unstimulated whole saliva flow rate ≤ 0.1 mL/min. The first two items have the highest weights, 3 points each, and the last three items a weight of 1 point each. A diagnosis of primary SS is tenable when the total score is 4 or more [8]. The combination of RF and ANA is no longer included in the criteria, and anti-SSB positivity has been removed from the criteria since positive anti-SSB in the absence of anti-SSA has no significant association with SS phenotypic features [8, 9]. Regardless of which set of criteria is used, a patient with dry eye and/or dry mouth findings but negative serology must undergo a minor salivary gland biopsy to confirm the diagnosis. Current traditional serological markers are limited in their utility with high rates of false negative results. For instance, anti-SSA antibodies are detected in only about 33-74% of patients with SS [10]. Thus, there is a need for better serological markers.

Various autoantibodies have been investigated regarding their utility in the diagnosis of SS. [11–13] Three novel autoantibodies, anti-salivary gland protein 1 (SP1), anti-carbonic anhydrase 6 (CA6), and anti-parotid secretory protein (PSP), have been suggested as useful markers to identify patients who are in the early stages of SS and perhaps with negative traditional antibodies [14, 15]. Although these novel autoantibodies were initially discovered in a mouse model, previous studies have explored their potential utility in humans [15–17]. We recently evaluated these antibodies in a small sample of dry eye patients in a prospective cross-sectional study and found that anti-CA6 was associated with severe aqueous-deficient dry eye indicating perhaps an early SS without positive traditional antibodies [17]. In this current study, a larger pool of dry eye patients with clinically significant aqueous tear deficiency who were suspected of having a systemic inflammatory/autoimmune disease and underwent a full battery of diagnostic testing was reviewed.

## 2. Materials and Methods

### 2.1. Patients

This retrospective study was approved by the Johns Hopkins University Institutional Review Board, and the study protocol adhered to the Health Insurance Portability and Accountability Act and the tenets of the Declaration of Helsinki. Patients who were examined at the Ocular Surface Diseases and Dry Eye Clinic, the Wilmer Eye Institute, the Johns Hopkins University School of Medicine, Baltimore, Maryland, with a primary diagnosis of dry eye and underwent serological testing for novel autoantibodies were considered for inclusion. The recommendation of serological testing for SS is based on the severity of dry eye (OSS ≥ 3 and/or Schirmer test ≤ 5 mm) or positive review of system items suggesting the presence of underlying systemic disease such as dry mouth, joint/muscle pain, or fatigue. In this analysis, patients with a primary diagnosis of other ocular diseases such as sterile keratitis, scleritis, or uveitis were not included. Patients having a recent history of ocular surgery, use of certain medications, or contact lens wear were not excluded. Nevertheless, none of the patients had a recent history of any ocular surgery. Six patients were on topical glaucoma medications, and none was a current contact lens wearer. A patient list was electronically generated using the test code QX206/T5307 for “Early Sjögren's Profile” (LAB26196) between April 2014 and July 2017. Medical records of patients were reviewed retrospectively. All available information was collected from each patient's chart regarding demographics; dry eye-related symptoms; SS-related systemic symptoms such as dry mouth, joint/muscle pain, fatigue, diagnosis of SS, and other associated autoimmune diseases; clinical signs of dry eye; serological test results; and minor salivary gland biopsy findings.

### 2.2. Evaluation of Dry Eye

Patients were evaluated by a single ophthalmologist (EKA) in a uniform manner. A complete medical history and review of systems were performed first. Dry eye was assessed using tear osmolarity, Schirmer test, and ocular surface staining, in the order mentioned here. Tear osmolarity was measured using the TearLab Osmolarity System (TearLab Corporation Inc., San Diego, CA) according to the manufacturer's recommendations [18]. The Schirmer test was performed without topical anesthesia using sterile strips (TearFlo, Sigma Pharmaceuticals, Monticello, IA), and the amount of paper wetting was measured in mm after 5 minutes. Ocular surface staining was performed using lissamine green dye for the conjunctiva (GreenGlo, HUB Pharmaceuticals LLC., Rancho Cucamonga, CA) and fluorescein for the cornea (Ful-Glo, Akorn Inc., Lake Forest, IL). Corneal staining was evaluated using a cobalt blue filter, and conjunctival staining was evaluated using a neutral density filter. Ocular surface staining score was calculated for the cornea and conjunctiva separately and then summed for a total OSS for each eye according to the Sjögren's International Collaborative Clinical Alliance (SICCA) grading system [19]. The maximum possible corneal staining score was 6 (the punctate epithelial erosions grade between 0 and 3 plus any extra points for modifiers such as central corneal staining, confluent staining, and filaments). Nasal and temporal conjunctiva staining was graded separately with a maximum score of three for each area, for a total of 6. The maximum possible OSS was 12 for each eye [19].

### 2.3. Laboratory Tests

Venous blood samples of patients were collected at the Johns Hopkins Medical Laboratories for serological testing. Testing for RF, ANA, and antibodies to SSA and SSB was performed at the Johns Hopkins Medical Laboratories. Additional venous blood samples were sent to the IMMCO Diagnostics Lab (Buffalo, NY) for serological testing for novel autoantibodies (SP1, CA6, and PSP). The presence of IgG, IgA, and IgM antibodies to SP1, CA6, and PSP was each reported individually. Whenever testing for any of the three isotypes was above the normal range, the result was considered positive. The cutoff for positivity for ANA was 1 : 320.

### 2.4. Statistics

The worse eye values for dry eye measures (higher osmolarity, lower Schirmer's value, and higher OSS) were used for the data analysis. The *t*-test and analysis of variance (ANOVA) were used to compare the continuous variables, and the chi-square test was used to compare categorical variables between groups. Spearman's rank correlation coefficient was used to analyze the association between a continuous and binary variable, and the phi coefficient was used to analyze the association between two binary variables. Logistic regression models were used to quantify the associations between serological markers and clinical measures after adjustment for potential confounders such as age, sex, and other serological markers. Values of *p* < 0.05 were considered statistically significant. All statistical analyses were performed using IBM SPSS Statistics version 23 (IBM Corp., Armonk, NY).

## 3. Results

One hundred and thirty-six patients with a primary diagnosis of dry eye underwent serological testing for novel autoantibodies between April 2014 and July 2017 and were included in the analysis. Nine of these patients had a prior patient-reported history of SS at the time of the testing, 5 primary SS and 4 secondary (3 with RA and 1 with mixed connective tissue disease). Testing was repeated to confirm the diagnosis in these patients. Fourteen patients were previously tested and had negative results, and the testing was repeated based on high clinical suspicion. The remaining 113 patients had never been tested for SS before. Of the 113 patients, 12 had a known history of inflammatory systemic disease (RA = 7, psoriasis = 4, and seronegative spondyloarthropathy = 1). As a result of the initial evaluation, 9 patients (9/127, 7%) received a new diagnosis of SS, 6 primary SS and 3 secondary SS (2 with RA and 1 with psoriatic arthritis). All but one received a new diagnosis of SS based on positive serology. The only seronegative patient needed to undergo a lip biopsy to be classified as having primary SS.

The mean age of patients was 59.8 ± 11.7 years, and a greater proportion of patients were female (85%) with a female-to-male ratio of 5 : 1. The most commonly reported dry eye-related symptom was foreign body sensation/grittiness (91/136, 67%), followed by burning/tearing (70/136, 51%), light sensitivity (55/136, 40%), blurred vision (52/136, 38%), and eye pain (43/136, 32%). Fifty-six patients had at least one SS-related extraocular symptom (dry mouth, joint/muscle pain, or fatigue), with dry mouth being the most commonly reported symptom (43/136, 32%). The average value of tear osmolarity was 308 ± 18 mOsm, Schirmer test was 7.8 ± 7.2 mm, and OSS was 6.2 ± 3.4 (cornea score, 2.6 ± 1.8, and conjunctiva score, 3.6 ± 2.2). Thirty-nine patients (29%) had Schirmer test score ≤ 5 mm, 83 patients (61%) had OSS ≥ 5, and 100 patients (73%) had either Schirmer test ≤ 5 mm or OSS ≥ 5.

**Figure 1:**
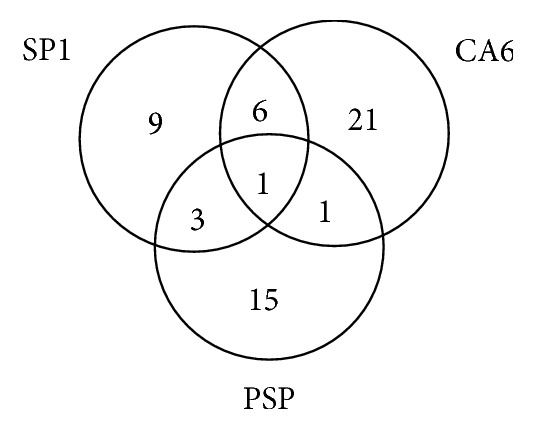
Venn diagram showing the number of patients with positive novel autoantibody.

Fifty-six patients (56/136, 41%) tested positive for at least one of the novel autoantibodies, 21 had anti-CA6 alone, 15 had anti-PSP alone, 9 had anti-SP1 alone, 6 had both anti-SP1 and anti-CA6, 3 had both anti-SP1 and anti-PSP, 1 had both anti-CA6 and anti-PSP, and 1 had all three of them (Figure 1).

With regard to demographic or clinical characteristics, no significant difference was found between patients with positive versus negative novel autoantibodies as shown in Table 1.

Table 2 displays demographic and clinical characteristics of patients according to autoimmune disease status including patients who had a known diagnosis of SS prior to the testing as well as those who received a new diagnosis of SS based on the test results.

Novel autoantibodies were detected in 8 of the 11 patients with primary SS (73%), 1 of the 7 patients with secondary SS (14%), and 10 of the 21 patients with other autoimmune diseases in the absence of SS diagnosis (48%). In addition, 37 of the 97 (38%) patients with no known autoimmune diseases at the time of the testing were positive for at least one novel autoantibody. Anti-PSP was the most frequently detected novel autoantibody in patients with primary SS (6/8, 75%), while anti-CA6 was the most commonly detected novel autoantibody in patients with other autoimmune diseases (5/10, 50%) and with no known autoimmune diseases (21/37, 57%). Two of the 11 patients with primary SS were seronegative, and both tested positive for anti-PSP.

Anti-PSP was the only novel autoantibody that correlated with having primary SS (*ɸ* = 0.33, *p* < 0.001). Correlations between the antibody status and the severity of dry eye measures are shown in Table 3. Based on logistic regression analysis after adjustment for age, sex, and other serologic markers, anti-CA6 showed a significant association with both corneal and conjunctival staining scores (HR = 1.5, 95% CI = 1.20-1.97, and *p* = 0.009 and HR = 1.4, 95% CI = 1.04-1.76, and *p* = 0.02, respectively), and ANA showed a significant association with corneal staining score (HR = 1.7, 95% CI = 1.04-2.65, and *p* = 0.03). (Table 4).

## 4. Discussion

This retrospective study evaluated the clinical relevance of the three novel autoantibodies, anti-SP1, anti-CA6, and anti-PSP, in patients with clinically significant aqueous-deficient dry eye who were suspected of having an underlying autoimmune disease, particularly SS. Our results demonstrate that anti-PSP was the most prevalent of the novel autoantibodies in patients with primary SS (6/11, 54%). Also, anti-PSP was detected in 2 seronegative SS patients with definitive diagnosis. On the other hand, anti-CA6 was the most prevalent of the novel autoantibodies in patients without any known autoimmune disease and the only novel autoantibody associated with severe ocular surface staining (both corneal and conjunctival). These findings are consistent with our previous report [17] and support the theory that anti-CA6 may be a marker indicating early stages of SS or another form of an autoimmune dry eye.

Inflammation regulated by both innate and adaptive immune systems plays a crucial role in ocular surface damage due to SS-related dry eye [2]. Although not fully known, an adaptive immune response to autoantigens is thought to be the triggering mechanism in SS [20]. Thus, understanding the role of autoantigens in the pathogenesis will influence the diagnosis and management of the disease. Antibodies to SSA antigens, components of a ribonucleoprotein complex, were the most commonly detected antibodies in patients with SS and currently the only serologic marker included in the most recent classification criteria for SS [8]. Other autoantibodies have been implicated in playing a role in the pathogenesis of SS, but none are currently included in the diagnostic criteria [20]. Three antigens, SP1, CA6, and PSP, are selectively expressed in salivary and lacrimal glands as opposed to the SSA antigen that can be expressed in any cell with a nucleus [15]. In fact, anti-SSA antibodies can be detected in other autoimmune diseases such as systemic lupus erythematosus or primary biliary cirrhosis without coexistent SS [21]. The animal models of SS suggest that SS starts as an organ-specific disease [14]. Therefore, detecting antibodies to salivary and/or lacrimal gland-specific antigens may, in fact, indicate early stages of the disease.

In our study, anti-PSP antibodies were detected more prominently in patients with primary SS including two patients who were seronegative at the time of the testing. PSP is one of the major secretory proteins of the parotid gland and functions as an antimicrobial agent to protect tissue surfaces exposed to the external environment [22]. Its abnormal expression has been shown in the submandibular glands as well as in lacrimal glands of an animal model of autoimmune sialadenitis [22]. To the best of our knowledge, the presence of anti-PSP antibodies alone has not been previously reported in patients with an established diagnosis of primary SS. In a previous study, novel autoantibodies were investigated in the sera of patients from the SICCA cohort and patients were grouped according to their focus score determined from minor salivary gland biopsy as the indicator of disease severity [15]. Neither anti-SP1 nor anti-PSP was detected at a level that was significantly higher in any of the study groups [15]. In our study, the biopsy information was available in one of the two seronegative patients who tested positive for anti-PSP and focus score was determined as 3.4 per 4 mm^2^. Although the number of patients with SS was considerably smaller in our study (*n* = 18) and biopsy information was not available for the majority of patients, we believe that significance of anti-PSP in patients with primary SS should be further investigated in future studies with larger sample size.

Anti-CA6 deserves particular attention in the present study since it was the most prevalent of the novel autoantibodies. More importantly, anti-CA6 was more frequent in patients with no known autoimmune diseases at the time of the testing. Similarly, in our previous report, we detected anti-CA6 antibodies in 43% of the patients who had significant dry eye but negative serology and biopsy (thus not fulfilling 2012 ACR classification criteria for SS) [17]. Higher levels of anti-CA6 were also demonstrated in patients from the SICCA cohort who had significant dry eye and dry mouth but no lymphocytic focus [15]. Carbonic anhydrases are responsible for regulation of acid-base balance in both physiological and pathological states [23]. CA6 is the only secretory isoenzyme of the carbonic anhydrase enzyme family expressed by parotid and submandibular glands as well as lacrimal glands [23–25]. Cytosolic CA6 is responsible for electrolyte and water secretion by the acinar cells in both salivary and lacrimal glands. CA6 in the secretory granules, on the other hand, is discharged into the acinar lumen to maintain bicarbonate levels to regulate pH in tear fluid and protect the corneal and conjunctival epithelial cells against injuries [25]. The significant association that we found between anti-CA6 and severe ocular surface staining can be explained either by reduced secretion or by changes in pH of the tear film which make epithelial cells vulnerable. The latter makes more sense as there was no significant association between anti-CA6 and decreased tear volume. More studies are needed to explain these findings further.

Previous studies demonstrated increased levels of anti-SP1 antibodies in patients with SS, particularly in patients with secondary SS in the setting of RA [26]. Anti-SP1 antibodies were not notably prevalent in our study; however, only one in 7 patients with secondary SS tested positive for novel autoantibodies, which was anti-SP1, and this only patient had SS secondary to RA. SP1 is a murine protein expressed by both submandibular and lacrimal glands [27]. The human homolog of this protein was not known, but recently, human common SP1 was identified in the saliva of patients with periodontitis at higher levels compared to healthy individuals [23]. This protein is known to be highly expressed in stressed conditions and regulates the oral microflora through its antimicrobial activity [28]. In a recent study, anti-SP1 antibodies were predominantly detected in patients with the Schirmer test measured between 3 and 6 mm, while anti-CA6 antibodies were predominantly detected in patients with the Schirmer test less than 3 mm [29]. Since the recent classification criteria for SS require the cutoff value of 5 mm for the Schirmer test [8], we looked for correlations between the presence of certain antibodies and having the Schirmer test 5 mm or less. In our study, anti-SP1 was the only antibody correlated with having a Schirmer test ≤ 5 mm.

A recently published report on these novel autoantibodies in participants of the DREAM Study demonstrated a higher prevalence of anti-SP1 in patients with SS-related dry eye compared to patients with non-SS dry eye (33% vs. 19%) [14]. Of note, aside from using a different set of criteria for dry eye in the DREAM Study, it was not specified whether the SS group included patients with primary SS, secondary SS, or both. [14] In our study, the prevalence of anti-SP1 in patients with both primary and secondary SS was 22% while it was 13% in patients with non-SS dry eye. The most prevalent novel autoantibody in patients with both primary and secondary SS was still anti-PSP (33%).

We cannot stress enough the importance of a heightened suspicion for underlying autoimmune disease in patients with significant dry eye. As a result of the initial testing, we were able to newly diagnose nine additional patients (7%), 2 of whom tested negative previously. In a study from our clinic, 12 patients received a new diagnosis of SS which corresponded to 6% of the patients who were evaluated for an underlying SS [4]. These rates are in line with previous studies [5, 16]. Furthermore, the necessity of further evaluations in patients with previous negative workup has been emphasized in earlier reports [5]. In our study, 8 in 13 patients with a previous negative workup for SS tested positive for at least one novel autoantibody while no traditional autoantibody was positive. Anti-CA6 was the most prevalent of novel autoantibodies detected in these patients. If these antibodies are indicating early stages of SS, the evolution of the disease from an organ-specific level to a systemic disease can be explained by the secondary immune response theory. Autoantigens expressed in specific tissues are targeted by the antibodies, and damage releases other autoantigens triggering secondary immune responses which may sustain the autoimmune disease [30]. First antigens, as well as the antibodies against them, could disappear in time as a result of the complete destruction of targeted tissues at the beginning [20]. This could perhaps explain why some markers disappear in time while others appear later on. Alternatively, autoimmunity towards SSA antigens can be induced by a protein that shares a sequence [20]. Further studies can shed light on this theory to understand the roles of these antigens in the pathogenesis.

One of the limitations of this study is the retrospective nature of the data collection which may have caused information bias due to missing information or measurement error. Another limitation is the small number of patients with SS, resulting in lower statistical power for the analysis in this group. Since minor salivary gland biopsy information was not available for the majority of the patients, it is not known whether seronegative patients definitively did not have SS at the time of the testing. Further, it was not possible to determine who would eventually develop SS given a longer follow-up due to the cross-sectional design of this study. Therefore, our findings should be interpreted cautiously.

## 5. Conclusions

Uncovering an underlying inflammatory/autoimmune disease in patients with dry eye is clinically relevant. SS is highly prevalent in a dry eye population but frequently underdiagnosed due to not only underappreciation of the disease but also the complexity of the clinical findings and difficulties with the currently available diagnostic testing. Whether patients with positive novel autoantibodies represent early stages of SS or another type of autoimmune dry eye deserves longitudinal studies with larger sample size.

## Figures and Tables

**Table 1:** Demographic and clinical characteristics of patients according to novel autoantibody status.

	Novel antibody positive (*n* = 56)	Novel antibody negative (*n* = 80)	*p* value
Demographics			
Age, years, mean (SD)	59.0 (10.5)	60.4 (12.4)	0.50
Female, *n* (%)	49 (87%)	66 (82%)	0.48
Dry eye-related symptoms, n (%)			
Light sensitivity	22 (39%)	33 (41%)	0.86
Foreign body sensation/grittiness	39 (70%)	52 (65%)	0.58
Burning/tearing	27 (48%)	43 (54%)	0.60
Eye pain	18 (32%)	25 (31%)	0.99
Blurred vision	22 (39%)	30 (37%)	0.86
Dry eye measures, mean (SD)			
Tear osmolarity (mOsm/L)	309 (22.6)	307 (14.6)	0.61
Schirmer test (mm)	6.6 (5.7)	8.5 (7.8)	0.27
Schirmer test ≤ 5 mm, *n* (%)	19 (63%)	20 (43%)	0.07
Total OSS (0-12)	6.4 (3.3)	6.0 (3.5)	0.56
Corneal staining (0-6)	2.7 (1.8)	2.5 (1.9)	0.57
Conjunctival staining (0-6)	3.7 (2.0)	3.6 (2.3)	0.74
OSS ≥ 5, *n* (%)	35 (66%)	48 (67%)	0.94
Schirmer ≤ 5 mm or OSS ≥ 5, *n* (%)	42 (78%)	58 (76%)	0.84
SS-Related Symptoms, n (%)			
Dry mouth	18 (32%)	24 (30%)	0.85
Joint/muscle pain	12 (21%)	12 (15%)	0.37
Fatigue	6 (11%)	10 (12%)	0.79
Autoimmune Diseases			
Sjögren's syndrome	9 (16%)	9 (11%)	0.41
Primary	8 (14%)	3 (4%)	0.051
Secondary	1 (2%)	6 (7%)	0.24
Other autoimmune diseases	10 (18%)	11 (14%)	0.57
SS diagnostic parameters			
Anti-SSA	6 (11%)	7 (9%)	0.68
Anti-SSB	2 (4%)	3 (4%)	>0.99
RF	7 (13%)	7 (9%)	0.46
ANA ≥ 1 : 320	7 (13%)	7 (9%)	0.46
Positive biopsy	1/6 (17%)	1/7 (14%)	>0.99

Results are represented as mean (standard deviation) for continuous variables and number (percentage) for binary variables. The t-test was used for comparison of continuous variables and chi-squared testing for categorical variables between groups. SD: standard deviation; OSS: ocular staining score; SSA: Sjögren's syndrome-related antigen A; SSB: Sjögren's syndrome-related antigen B; RF: rheumatoid factor; ANA: antinuclear antibody.

**Table 2:** Demographic and clinical characteristics of patients according to autoimmune disease status.

	Primary SS (*n* = 11)	Secondary SS (*n* = 7)	Other autoimmune disease (*n* = 21)	No known autoimmune disease (*n* = 97)	*p* value
Demographics					
Age (years), mean (SD)	58 (8.4)	61 (15.5)	58 (15.3)	60 (10.9)	0.82
Female, *n* (%)	11 (100%)	7 (100%)	18 (86%)	79 (81%)	0.26
SS-related symptoms, n (%)					
Dry mouth	3 (27%)	2 (29%)	8 (38%)	30 (31%)	0.91
Joint/muscle pain	2 (18%)	0	2 (9%)	20 (21%)	0.38
Fatigue	2 (18%)	1 (14%)	1 (5%)	13 (13%)	0.67
Diagnostic parameters, n (%)					
Anti-SSA	9 (82%)	4 (57%)	0	0	
Anti-SSB	3 (27%)	2 (29%)	0	0	
RF	5 (46%)	4 (57%)	3 (14%)	2 (2%)	
ANA ≥ 1 : 320	6 (55%)	3 (42%)	3 (14%)	2 (2%)	
Positive biopsy	2/3 (67%)	0/1 (0%)	0/2 (0%)	0/7 (0%)	
Novel autoantibodies, n (%)	8 (73%)	1 (14%)	10 (48%)	37 (38%)	0.06
Anti-SP1	3 (27%)	1 (14%)	2 (9%)	13 (13%)	0.57
Anti-CA6	3 (27%)	0	5 (24%)	21 (22%)	0.53
Anti-PSP	6 (54%)	0	4 (19%)	10 (10%)	**0.001**
Dry eye measures, mean (SD)					
Tear osmolarity (mOsm/L)	323 (33.7)	308 (17.4)	307 (17.7)	307 (14.5)	0.054
Schirmer test (mm)	9.0 (8.1)	4.8 (3.6)	7.7 (11.5)	7.9 (6.7)	0.79
Schirmer test ≤ 5 mm, *n* (%)	5 (71%)	2 (67%)	7 (78%)	25 (43%)	0.14
Total OSS (0-12)	8.3 (3.3)	8.6 (1.8)	7.2 (2.7)	5.5 (3.5)	**0.003**
Corneal staining (0-6)	3.3 (1.8)	3.4 (1.5)	2.9 (1.9)	2.3 (1.8)	0.15
Conjunctival staining (0-6)	5.0 (1.8)	5.1 (1.2)	4.4 (1.6)	3.2 (2.3)	**0.002**
OSS ≥ 5, *n* (%)	6 (60%)	4 (67%)	13 (68%)	60 (72%)	0.97
Schirmer ≤ 5 mm or OSS ≥ 5, *n* (%)	8 (73%)	5 (83%)	17 (85%)	70 (75%)	0.77

Results are represented as mean (standard deviation) for continuous variables and number (percentage) for binary variables. The one-way analysis of variance (ANOVA) was used for comparison of continuous variables and chi-squared testing for categorical variables between groups. SS: Sjögren's syndrome; SD: standard deviation; SSA: Sjögren's syndrome-related antigen A; SSB: Sjögren's syndrome-related antigen B; RF: rheumatoid factor; ANA: antinuclear antibody; SP1: salivary protein 1; CA6: carbonic anhydrase 6; PSP: parotid secretory antigen; OSS: ocular staining score.

**Table 3:** Correlations between positive antibody status and dry eye measures.

		Anti-SSA	Anti-SSB	RF	ANA	Anti-SP1	Anti-CA6	Anti-PSP
Tear osmolarity	Rho	0.08	-0.06	0.05	0.03	-0.01	-0.08	0.05
	*p* value	0.39	0.52	0.63	0.80	0.92	0.40	0.60
Schirmer test	Rho	0.06	-0.01	-0.12	0.03	0.13	-0.06	-0.08
	*p* value	0.62	0.91	0.30	0.83	0.26	0.62	0.49
Corneal staining	Rho	**0.18**	**0.22**	**0.27**	**0.28**	-0.10	**0.21**	0.02
	*p* value	**0.04**	**0.01**	**<0.001**	**<0.001**	0.26	**0.02**	0.79
Conjunctival staining	Rho	**0.24**	0.13	**0.22**	**0.18**	-0.09	**0.19**	-0.03
	*p* value	**0.01**	0.15	**0.01**	**0.04**	0.31	**0.03**	0.72
Total OSS	Rho	**0.25**	**0.20**	**0.29**	**0.27**	-0.10	**0.26**	0.00
	*p* value	**0.01**	**0.02**	**<0.001**	**<0.001**	0.29	**<0.001**	0.97

Spearman correlation coefficient (rho) was used to analyze the associations between variables. Bolded values represent *p* < 0.05. SSA: Sjögren's syndrome-related antigen A; SSB: Sjögren's syndrome-related antigen B; RF: rheumatoid factor; ANA: antinuclear antibody; SP1: salivary protein 1; CA6: carbonic anhydrase 6; PSP: parotid secretory antigen; OSS: ocular staining score.

**Table 4:** Multiple regression analysis demonstrating associations of antibody positivity with clinical parameters.

	Anti-SSA	Anti-SSB	RF	ANA	Anti-SP1	Anti-CA6	Anti-PSP
Tear osmolarity (mOsm/L)	1.0 (0.95-1.05)	1.6 (0.00-1.552E+83)	1.0 (0.98-1.07)	1.0 (0.95-1.03)	1.0 (0.96-1.03)	1.0 (0.96-1.03)	1.0 (0.99-1.05)
Schirmer test (mm)	1.2 (0.99-1.49)	0.3 (0.00-.)	0.7 (0.40-1.17)	1.1 (0.94-1.28)	1.1 (0.98-1.21)	0.9 (0.84-1.05)	0.9 (0.77-1.06)
Corneal staining (0-6)	0.8 (45-1.60)	2.1 (0.00-1358.86)	1.8 (0.97-3.29)	**1.7 (1.04-2.65)**	0.7 (0.49-1.02)	**1.5 (1.20-1.97)**	1.1 (0.80-1.46)
Conjunctival staining (0-6)	1.3 (0.68-2.49)	0.5 (0.06-4.63)	1.3 (0.83-1.97)	1.1 (0.78-1.63)	0.8 (0.62-1.06)	**1.4 (1.04-1.76)**	1.0 (0.77-1.29)
Total OSS (0-12)	1.0 (0.71-1.50)	0.83 (0.16-4.40)	1.3 (0.96-1.77)	1.2 (0.96-1.60)	0.8 (0.67-0.99)	**1.3 (1.08-1.53)**	1.0 (0.88-1.24)

Each clinical measure was analyzed in a separate logistic regression model including each antibody as the dependent variable and the clinical measure, age, sex, and other serologic markers as independent variables. Values represent hazard ratios and 95% confidence intervals. Bolded values represent *p* < 0.05. SSA: Sjögren's syndrome-related antigen A; SSB: Sjögren's syndrome-related antigen B; RF: rheumatoid factor; ANA: antinuclear antibody; SP1: salivary protein 1; CA6: carbonic anhydrase 6; PSP: parotid secretory antigen; OSS: ocular staining score.

## Data Availability

The data used to support the findings of this study are available from the corresponding author upon request.
